# Rapid and High-Throughput Evaluation of Diverse Configurations of Engineered Lysins Using the VersaTile Technique

**DOI:** 10.3390/antibiotics10030293

**Published:** 2021-03-11

**Authors:** Lisa Duyvejonck, Hans Gerstmans, Michiel Stock, Dennis Grimon, Rob Lavigne, Yves Briers

**Affiliations:** 1 Laboratory of Applied Biotechnology, Department of Biotechnology, Ghent University, Valentin Vaerwyckweg 1, 9000 Ghent, Belgium; lisa.duyvejonck@ugent.be (L.D.); hans.gerstmans@kuleuven.be (H.G.); dennis.grimon@ugent.be (D.G.); 2 Laboratory of Gene Technology, Department of Biosystems, KU Leuven, Kasteelpark Arenberg 21, 3001 Leuven, Belgium; rob.lavigne@kuleuven.be; 3 MeBioS-Biosensors Group, Department of Biosystems, KU Leuven, Willem de Croylaan 42, 3001 Leuven, Belgium; 4 KERMIT and Biobix, Department of Data Analysis and Mathematical Modelling, Ghent University, Coupure links 653, 9000 Ghent, Belgium; michiel.stock@ugent.be

**Keywords:** lysin, bacteriophage, VersaTile, *Klebsiella pneumoniae*, protein engineering

## Abstract

Bacteriophage-encoded lysins are an emerging class of antibacterial enzymes based on peptidoglycan degradation. The modular composition of lysins is a hallmark feature enabling optimization of antibacterial and pharmacological properties by engineering of lysin candidates based on lysin and non-lysin modules. In this regard, the recent introduction of the VersaTile technique allows the rapid construction of large modular lysin libraries based on a premade repository of building blocks. In this study, we perform a high-throughput construction and screening of five combinatorial lysin libraries with different configurations, targeting *Klebsiella pneumoniae*. An elaborate analysis of the activity distribution of 940 variants and sequencing data of 74 top hits inhibiting the growth of *Klebsiella pneumoniae* could be associated with specific design rules. Specific outer membrane permeabilizing peptides (OMPs) and enzymatically active domains (EADs) are significantly overrepresented among the top hits, while cell wall binding domains (CBDs) are equally represented. Especially libraries with the configuration (OMP–linker–CBD–EAD) and the inverse configuration (CBD–EAD–linker–OMP) yield the most active variants, with discernible clusters of variants that emerge above the remaining variants. The approach implemented here provides a blueprint for discovery campaigns of engineered lysins starting from libraries with different configurations and compositions.

## 1. Introduction

Lysins are bacteriophage-encoded enzymes that degrade bacterial peptidoglycan. They include virion-associated peptidoglycan hydrolases (VAPGHs) and endolysins, which are essential in the process of phage genome injection and phage progeny release, respectively [1]. Based on their clinical impact and technical feasibility, they are regarded as one of the alternative classes with high potential as antimicrobial therapeutics for clinical practice [2]. This auspicious ability as potential therapeutics is attributable to a number of beneficial and unique properties associated with this class of antibacterials. First, lysins have a rapid mode-of-action based on enzymatic activity acting immediately upon contact. This active mechanism also enables the killing of persister cells [3]. Secondly, they possess a narrow-spectrum activity, hereby limiting the impact on the natural microbiome, in contrast to conventional broad-spectrum antibiotics. Thirdly, the development of bacterial resistance by the accumulation of point mutations is generally not observed for lysins [4]. In addition, initial concerns about the proteinaceous nature of lysins appear to be refuted. An antibody response is elicited but does not impede the in vivo activity, likely due to the rapid action of lysins. Also, allergic responses or other side effects are not reported in animals or clinical trials [1].

Apart from these properties, lysins differentiate from all other antibacterial classes in their modular composition and the associated opportunities to modulate the antibacterial properties. This modularity implies that lysins are composed of contiguous domains with distinct, dedicated functions, each affecting the antibacterial activity. These domains can be either a CBD or an EAD. Lysins can be engineered through recombining or swapping these domains from various sources to enhance their activity, solubility, stability, or specificity [1,5]. Moreover, the addition of non-lysin modules such as OMPs to transfer the lysin moiety through the otherwise lysin-impermeable outer membrane of Gram-negative bacteria (commercialized as Artilysin^®^) [6], cell-penetrating peptides to enter a eukaryotic cell [7], or an albumin-binding domain to increase the half-life of a lysin in blood, further expand the customization possibilities of lysins [8]. In this regard, libraries of increasing sizes are being screened to identify variants with the desired properties. Briers et al. (2014) constructed a library of 49 Artilysins with different configurations and evaluated the effect on the antibacterial activity against *Pseudomonas aeruginosa*, *Acinetobacter baumannii*, *Escherichia coli,* and *Salmonella enterica* serovar Typhimurium [6]. Hang et al. (2017) screened a library of 126 engineered lysins to identify active variants against planktonic and biofilm methicillin-resistant *Staphylococcus aureus* [9]. Further, Verbree et al. (2017) and Röhrig et al. (2020) screened a library of 174 and 322 variants of engineered lysins and other recombinant peptidoglycan hydrolases for activity against *Staphylococcus aureus* under raw milk and intracellular conditions, respectively [7,10]. These libraries of modular or truncated variants were all constructed with traditional restriction/ligation techniques, which are time-consuming and labor-intensive. To eliminate this technical hurdle, we have recently introduced a rapid DNA assembly method for an efficient combinatorial assembly of lysin domains, coined “the VersaTile technique”. With this approach, large libraries of combinatorically shuffled lysins comprising up to thousands or millions of variants can be created rapidly, starting from a practically infinite number of non-homologous domains [5].

Gerstmans et al. (2020) generated a library of around 10,000 shuffled lysins with the VersaTile technique and selected a variant with high inhibitory activity against the Gram-negative critical priority pathogen *Acinetobacter baumannii* under human serum conditions. To achieve this goal, they implemented iterative rounds in which repeatedly smaller and enriched libraries were created and tested, depending on the observed structure–activity relationships. Based on prior knowledge, they focused on a single configuration (OMP–linker–CBD–EAD) [5]. A linker was introduced to grant sufficient autonomy and flexibility to the OMP and CBD-EAD moieties. In this study, we applied VersaTile to establish a pairwise comparison of the influence of the modular configurations on the growth inhibitory activity of engineered lysins against *Klebsiella pneumoniae*. Four new configurations were generated and screened along with the previous configuration. In total, over 444,000 variants were constructed with the VersaTile technique, of which 940 variants could be screened using plate-based growth inhibitory assays.

We selected *Klebsiella pneumoniae* as a target species, an important ESKAPE pathogen (*Enterococcus faecium*, *S. aureus*, *K. pneumoniae*, *A. baumannii*, *Pseudomonas aeruginosa* and *Enterobacter* species). This pathogen ranks among the most urgent and serious threats for our healthcare system [11], as they have developed increased levels of resistance to commonly used antibiotics as well as last resort antibiotics. Indeed, *K. pneumoniae* strains producing extended-spectrum beta-lactamases (ESBLs) are increasingly prevalent or have evolved to carbapenem-resistant *Enterobacteriaceae* (CRE) [12].

## 2. Results

### 2.1. The Construction of Five Libraries with Different Configurations Using VersaTile

**Figure 1:**
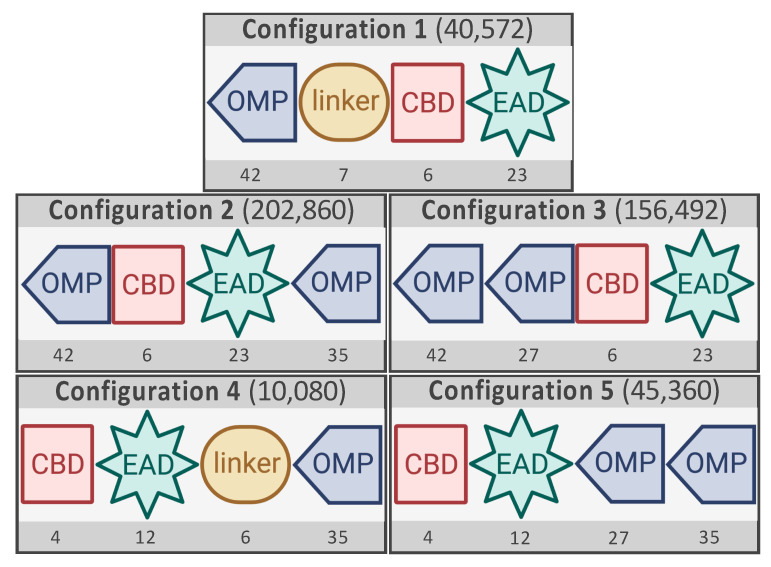
The different configurations tested in this study. Configuration 1 is the configuration analyzed in previous work [5]. OMP: outer membrane permeabilizing peptide, CBD: cell wall binding domain, EAD: enzymatically active domain. The total number of available tiles for configurations 1 through 5 are mentioned under each specific position. The resulting possible variants are indicated within the brackets.

The VersaTile technique entails two steps. First, a repository of building blocks (called tiles) needs to be constructed. A tile comprises the coding sequence and is flanked by position tags, labeling its position in the final assembly. Second, these tiles can be recombined in either a rational or combinatorial way. In a four-way system, at least one tile needs to be added for each of the four positions. Gerstmans et al. (2020) prepared a repository of 67 tiles in total (38 OMPs, 2 linkers, 6 CBDs, and 21 EADs at position 1, 2, 3, and 4, respectively) for the construction of a library with an OMP-linker-CBD-EAD configuration [5]. In this work, we constructed libraries with four additional configurations by repositioning the same defined modules and/or doubling the OMP module (Figure 1). As such, the impact of the configuration on the antibacterial activity can be assessed and specific design rules can be deduced.

All configurations were derived from the previously analyzed configuration 1 (OMP–linker–CBD–EAD). First, the combination CBD–EAD is retained in all configurations since this order mainly occurs in natural modular lysins produced by bacteriophages infecting Gram-negatives [6]. Next, the position of the OMP was altered. A single OMP was placed either at the N-terminal position (configuration 1) or the C-terminal position (configuration 4). Double OMPs were placed either at the N-terminus (configuration 3), at the C-terminus (configuration 5), or one at both sides (N- and C-terminus, configuration 2). In the case of a single OMP, a linker was included to specifically separate the OMP from the CBD–EAD domain, while conveniently retaining the four-way assembly setup present in the other configurations. To construct libraries with these four new configurations, the tile repository from our previous study was further expanded with additional tiles: (1) Tiles with the same coding sequences but different position tags; (2) Tiles with additional coding sequences for four OMPs, five linkers and two EADs with position tags for their respective positions. The previous repository only comprised two linker tiles encoding highly flexible linkers forming a coil solely consisting of small flexible amino acids (glycine and alanine). Therefore, additional linkers with a rigid structure were generated and included. This rigid structure can be achieved either through a helix or a proline-rich coil [13]. As such, the influence of the linker structure on the activity could also be evaluated. The total number of tiles was 218 (Figure 1; Supplementary Materials Table S2). A detailed description of each tile is given in Table S3. Note that the repository was partially asymmetric with not all coding sequences at each position. However, a minimum identical set of 27 OMPs, six linkers, four CBDs, and 12 EADs was available for each relevant position.

Based on the available repository, a combinatorial assembly reaction was performed for each configuration. The resulting complexities (Figure 1) are 40,572 (Configuration 1; 42 × 7 × 6 × 23), 202,860 (Configuration 2; 42 × 6 × 23 × 35), 156,492 (Configuration 3; 42 × 27 × 6 × 23), 10,080 (Configuration 4; 4 × 12 × 6 × 35), and 45,360 (Configuration 5; 4 × 12 × 27 × 35). This illustrates the high-throughput capacity of the VersaTile reaction. Previous deep sequencing experiments using Nanopore technology demonstrated equal incorporation of the variable tiles in the library variants [5,14].

### 2.2. The Hit Rate of the Libraries Depends on the Configuration of the Engineered Lysins

To evaluate and to compare the antibacterial activities of the variants from the different libraries, 188 variants were randomly selected from each configuration and expressed, followed by the preparation of cleared lysates. These cleared lysates were evaluated for their growth inhibitory activity against K. pneumoniae ATCC 13,833 both in the absence and the presence of 0.5 mM EDTA. EDTA chelates the divalent cations Ca^2+^ and Mg^2+^ and destabilizes the outer membrane since these cations are essential for outer membrane stability by bridging the phosphate groups of adjacent lipopolysaccharide molecules [15]. As such, EDTA may enhance the antibacterial activity [16] and may lower the detection limit of the assay, resulting in a higher number of hits. To compare the antibacterial activity and protein yield of the evaluated variants, we converted all values to growth inhibition percentages. A higher growth inhibitory percentage corresponds to a higher growth inhibitory activity.

**Figure 2:**
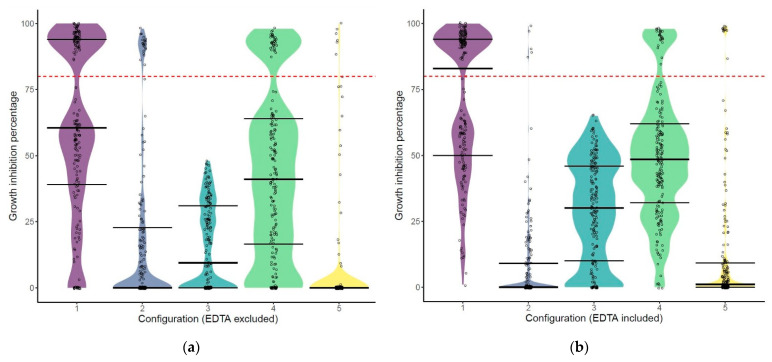
The distribution of the growth inhibitory (GI) activities, expressed as percentages, for each configuration. (**a**) The replicates tested in the absence of 0.5 mM EDTA. (**b**) The replicates tested in the presence of 0.5 mM EDTA. The red dashed line represents the GI threshold set in this study to be considered a hit (80%). The first configuration (purple) shows the most active variants. Configurations 1 and 4 have a top cluster with strong active variants, whereas configuration 2 and 5 show a long tail of active variants with rising activity. EDTA does not significantly alter the specific distribution of the variants, but slightly increases the activity for configurations 1, 3, 4, and 5.

Figure 2 shows the distribution of the growth inhibition percentages of the analyzed variants per configuration, in the absence (Figure 2a) or the presence (Figure 2b) of 0.5 mM EDTA. Two phenomena can be distinguished in the distribution of the activities. First, in the case of the best configurations (1 and 4), we see discernible top clusters of highly active variants and more stretched clusters of semi-active variants. Configuration 1 has the largest top cluster and represents as such the most successful combination of the available building blocks. The bulk of variants of configuration 2 and especially configuration 5 are chiefly grouped at the bottom with no inhibitory effect. However, they both show a long tail of variants with rising activity with a small differentiated top cluster. Configuration 3 shows a more equal distribution of active variants, but only up to a maximal inhibitory value of approximately 60%. Based on these findings, we will consider variants that inhibit the bacterial growth for more than 80% as hits.

When we look in more detail, we can state that screening four of five configurations resulted in numerous hits. Configuration 1 (OMP–linker–CBD–EAD) and configuration 4 (CBD–EAD–linker–OMP), which have a mirrored composition, have the highest hit rate (without EDTA: 38.3% and 18.6%, with EDTA: 50.5% and 12.8%, respectively). The hit rate for configuration 2 (OMP–CBD–EAD–OMP; without EDTA: 11.7%, with EDTA: 2.7%) and configuration 5 (CBD–EAD–OMP–OMP; without EDTA: 3.7%, with EDTA: 5.3%) is considerably lower, and all hits behave as outliers compared to the bulk of the variants. Notably, variants from configuration 3 that start with a double OMP (OMP–OMP–CBD–EAD) are active but do not inhibit the bacterial growth for more than 80% (hit rate of 0%). Beyond these differences in hit rate, EDTA has a positive impact on the antibacterial activity of all configurations except configuration 2. EDTA increases the growth inhibition percentage for these configurations by 12.9%, on average. In particular, EDTA has the most positive influence on the most performant configuration (configuration 1, +22%) and has the least effect on the antibacterial activity of the variants of configuration 5 (+1%).

### 2.3. Sequencing of the Most Active Hits Gives Design Rules

By sequencing the most active variants, insights are obtained on which tiles contribute the most to the growth inhibitory activity. This enables us to define structure–activity relationships, which in turn can serve as a starting point for improving or developing new libraries. Here, we sequenced the hits of the top clusters of the two most active configurations (configuration 1 and 4), specifically those hits with a growth inhibition percentage above 95%. For configuration 1, 53 different sequences were analyzed (Table S4), of which 33 reach a growth inhibition of 95% in the absence of EDTA and 36 reach 95% in the presence of EDTA. When variants inhibit more than 95% only in the absence of EDTA, the range of inhibition in the presence of EDTA is between 24–94%, whereas for variants that inhibit more than 95% only in the presence of EDTA, the range of inhibition in the absence of EDTA is between 0–91% (Figure S1). In the case of configuration 4, 21 different sequences were analyzed (Table S5), of which 12 and 15 reach a growth inhibition of 95% in the absence of and presence EDTA, respectively. When variants inhibit more than 95% only in the absence of EDTA, the range of inhibition in the presence of EDTA is between 38–94%, whereas for variants that inhibit more than 95% only in the presence of EDTA, the range of inhibition in the absence of EDTA is between 15–93% (Figure S2).

The different distributions per position and the occurrences of the different tiles at a given position were compared to an expected modelled multinomial distribution to assess if they differ significantly or not [14]. Analysis of the four types of building blocks shows that the distribution of OMPs deviates significantly from a uniform distribution (*p* < 0.1) for both configurations, both in the absence or the presence of EDTA (Table 1). Additionally for configuration 1, we see a significantly deviating distribution for EADs, regardless of the presence of EDTA, whereas, for configuration 4, this is also the case for the linkers in the presence of EDTA.

Therefore, we analyze the occurrences of the different, significantly deviating tile types. The overrepresented tiles are listed per position in Table 2.

The most common peptide in the first configuration on position 1 is OMP3 or Protegrin. This peptide is significantly overrepresented either with or without the addition of EDTA. More specifically, it is present in 21% (7/33) of all hits without EDTA and in 28% (10/36) hits in the presence of EDTA. Since the theoretical occurrence in the library is about 2.4%, this corresponds to a roughly tenfold overrepresentation. However, this peptide is not detected in the top hits of configuration 4 on position 4, where OMP5 or the Cathelicidin-BF antimicrobial peptide is most present. It is represented in 42% (5/12) in the hits without EDTA and 33% (5/15) in hits with EDTA, which is 20-fold more than the theoretical occurrence of 2.9% (1/35). This implies that tile overrepresentation is position-dependent in the configuration of the lysins.

Besides OMP3, OMP20 or Chrysophsin-1 also shows a significant positive influence on the antibacterial activity in the absence of EDTA, when assembled on position 1 of configuration 1. Here, it is represented in 12% (4/33) of the hits, which corresponds to a roughly 5-fold overrepresentation. Notably, both peptides do not show similar properties.

At the fourth position of configuration 1, EAD12 or the EAD of the modular 201ϕ2-1gp229 endolysin [16] is significantly overrepresented in the absence of EDTA, while EAD19 of the structural lysin of phage ϕKZ (KZgp181) [17] is significantly overrepresented in the hits identified in the presence of EDTA. Since the theoretical occurrence in the library for the EADs was about 4.3% (1/23), these tiles are almost 3.5 to 4.5-fold overrepresented (15% (5/33) and 19% (7/36), respectively). However, these EADs were not available on position 2 in the tile repository and were thus not evaluated in configuration 4.

At the third position of configuration 4, we see a 2-fold overrepresentation of the rigid long linker with a helical structure (Linker 4) (33%, 5/15) in the presence of EDTA, compared to its theoretical occurrence of 17% (1/6).

We also analyzed whether variants with a flexible linker perform significantly better or worse than variants with a rigid linker, but no difference was observed (No EDTA: *p* = 0.186 (configuration 1) and *p* = 0.122 (configuration 4), EDTA: *p* = 0.225 (configuration 1) and *p* = 0.301 (configuration 4)).

## 3. Discussion

VersaTile is a high-throughput DNA assembly method that enables the creation of a practically infinite number of shuffled and engineered lysins, starting from a repository comprising distinct building blocks, called tiles. A major advantage of this technique is the possibility to easily and quickly expand these repositories with new tiles and add to the availability of previously constructed tiles. Moreover, VersaTile addresses the need of continuously evolving research questions and the investigation of new design rules for these antibacterial enzymes. In this study, the total number of tested variants was increased by almost 2-fold in comparison with the highest number in previously published studies (940 versus 568 [5]). Nevertheless, this number of screened variants still represents low percentages of the whole libraries, with complexities ranging from 10,080 variants (188/10,080 = 1.87%) to 202,860 variants (188/202,860 = 0.0927%), highlighting the need for techniques that allow more representative high-throughput screenings. Although the screened variants are a randomly selected subset of the libraries, this subset gives an insightful snapshot of the different configurations. It turned out that the previously analyzed configuration is the configuration that yields the most active variants against *K. pneumoniae* ATCC 13883. Configuration 4 (the opposite combination) shows a smaller group of active variants as well. However, when implementing two peptides in the configuration (2, 3 and 5), we observe a strongly reduced number of active variants, especially when two OMPs are located at the N-terminus (configuration 3). A possible explanation for the inferior performance of the configurations with two peptides might be a reduced protein expression or inferior folding, especially in the case of random coils having little structure. Alternatively, double OMPs may result in an increased formation of aggregates due to the hydrophobic nature of some peptides, resulting in a smaller portion of active protein. Conversely, the higher activity of configuration 1 and 4 could be a result of the introduction of a linker between the OMP and the CBD–EAD moiety. This linker may grant more autonomy to both parts that must operate successively during the antibacterial action by introducing a physical separation. The lack of a linker in configuration 2, 3, and 5 may consequently inhibit this sequential action and autonomy of both moieties. Flexible linkers provide the lysins with a certain degree of conformational mobility between the functional domains, whereas rigid linkers rather fix the distance and relative orientation. It has also been reported that flexible linkers cause certain limitations, such as a poor protein expression or a reduction in biological activity. Rigid linkers are more appropriate to conserve the autonomous functions of the domains due to a more effective, physical separation [13]. Therefore, rigid linkers might improve the activity more than flexible linkers. However, in this study, we did not observe a significant overrepresentation of the rigid linkers compared to the flexible linkers. Hits were observed either without EDTA, with EDTA or in both conditions. This may imply that the variants are operating by different working mechanisms. Some may independently break through the OM locally by displacing the divalent ions from their binding site on the LPS, whereas others require EDTA to perform this action. The latter variants may have good characteristics to pass through the hydrophobic part (lipid A moiety and phospholipids) of the OM. In previous studies, EDTA significantly improved the antibacterial activity of lysins against *P. aeruginosa* and *A. baumannii*. However, when targeting the *Enterobacteriaceae* members *E. coli* and *S. enterica* serovar Typhimurium, this effect was not clearly observed [3,6]. Also in this study, we only observed a limited effect of EDTA against the *Enterobacteriaceae* member *K. pneumoniae*. The difference in affecting the antibacterial activity may be related to the differences of the outer membrane structures of these bacteria. The phosphate-rich outer membrane of *P. aeruginosa* and *A. baumannii* correlates with a higher amount of divalent cations such as Mg^2+^ and Ca^2+^ that bridges adjacent LPS molecules and stabilizes the outer membrane. *Enterobacteriaceae* contain a lower phosphate content, but instead, the stabilization occurs through hydrophobic interactions between the longer acyl chains of the lipid A moiety [18]. It should be noted that EDTA cannot be used for systemic use because of its anticoagulant properties [19] and was mainly evaluated here to improve the sensitivity of the assay.

We have shown here that the VersaTile technique allows to set up quick screenings of an unprecedented number of variants, by eliminating the hurdle of library construction. However, this brings up new challenges in library screening as a practically infinite number of choices can be made at this stage. The first law of directed evolution states ‘you get what you screen for’ [20]. Therefore, the choice of an assay, screening conditions and test species affect the eventual outcome. Röhrig et al. [7] demonstrated a high inter-assay variability, comparing time-kill, turbidity reduction, and spot on the lawn assays under extracellular and intracellular conditions. The authors mediated these biases by performing multiple assays and integrating all outputs in a weighted score. The challenge is thus to define robust and relevant in vitro proxy assays for the envisioned in vivo applications. The screening conditions can be either general as performed in this study or can be tailored to the conditions prevailing in the natural infection habitat of *Klebsiella* species, such as human serum, urine, and lung fluid. The same screening assays can be performed using artificial media that mimic the actual growth conditions such as synthetic urine or synthetic lung fluid. The inclusion of several strains having different bacterial capsules and LPS compositions will also strengthen the robustness of the extracted design rules. Such multiparametric screening campaigns require extensive efforts and are facilitated by dedicated equipment such as an automated liquid handler.

The screening was performed using cleared lysates instead of purified proteins to enable dealing with a large number of variants (as well as reduce cost). This implies that the screening outcome is influenced by the expression yield, protein solubility and growth inhibitory activity per mole. While screening with cleared lysates is a standard approach in protein engineering, this hinders the separate evaluation of these factors. Yet, from the perspective of downstream applications, the combination of a high production yield of soluble protein with high antibacterial activity is the most desired outcome and these variants will eventually prevail in the followed screening setup. Indeed, in our previous study, the lead molecule selected after three iterative screenings combined a high expression yield and antibacterial activity [5]. The current screening setup and stringency also resulted in a clear differentiation of variants between and within the configurations, indicating a good resolving power to rank configurations and variants and to learn design rules.

## 4. Materials and Methods

### 4.1. Bacterial Strains and Growth Media

For the storage of the plasmids necessary for the VersaTile reaction, *Escherichia coli* TOP10 cells were chosen, whereas for the expression of the proteins, we used *E. coli* BL21-CodonPlus(DE3)-RIL cells (Agilent Technologies, Belgium). Both strains were grown either in Lysogeny Broth (LB) (1% (*w*/*v*) tryptone, 0.5% (*w*/*v*) yeast extract, 1% (*w*/*v*) NaCl) and shaking at 180 rpm and 37 °C, or on LB agar (addition of 1.5% (*w*/*v*) bacteriological agar) at 37 °C. For small-scale expression of the lysin variants, *E. coli* cells were grown in auto-induction medium. This medium consists of a combination of ZY medium, 2 M MgSO_4_, 50× 5052-solution, and 20× NPS [16]. To select for plasmid-containing *E. coli* cells, media were supplemented with either 100 µg/mL ampicillin or 50 µg/mL kanamycin, and 5% (*w*/*v*) sucrose. For the high-throughput antibacterial assay, *Klebsiella pneumoniae* strain ATCC 13883 was grown in Mueller Hinton (MH) broth.

### 4.2. Expanding of Tile Repository: VersaTile Cloning

The previously reported tile repository [5] was expanded with new tiles and tiles with altered positions. The construction of new tiles (linkers) in this study was performed by hybridization of partially overlapping primer cassettes (Table S1; Integrated DNA Technologies, Leuven, Belgium). To alter the positions of existing tiles, a PCR amplification with tailed primers was performed using previously made tiles (in their entry vectors) as a template. The tailed primers consisted of three parts. The 5′ end contained a SapI recognition and restriction site for cloning in the entry vector pVTE (VersaTile cloning). Next, a BsaI recognition and restriction site within an appropriate position marker was included for the assembly of the four building blocks (VersaTile shuffling). Last, a sequence complementary to the coding sequence of the respective tile was placed at the 3′ end of the primer.

PCR amplification of the new tiles was performed using Pfu DNA polymerase (1.25 U) according to the manufacturer’s guidelines. For the hybridization of the primer cassettes, a fill-in reaction was needed. First, an equal ratio of the primers (10 μM) was mixed and incubated for 2 min at 95 °C. Next, to form dimers, the reaction was gradually cooled down until room temperature. Subsequently, the fill-in reaction of the single overhanging strands was performed using Pfu DNA polymerase (1.25 U, 10 min at 72 °C).

The reaction mixture for the simultaneous restriction and ligation of the amplified fragments and the filled-in primer cassettes in the entry vector pVTE3, contained 2 μL T4 DNA ligase buffer, 3 U T4 DNA ligase, 100 ng pVTE3, 50 ng of the amplicon/primer cassette, and 10 U SapI, to a total volume of 20 µL. After ligation, the mixture was used for transformation of chemically competent *E. coli* TOP10 cells, followed by selection on LB 1.5% agar with 100 µg/mL ampicillin and 5% (*w*/*v*) sucrose. Last, the tile entry vectors were isolated by plasmid extraction and the sequence was confirmed using Sanger sequencing (LGC Genomics, Berlin, Germany). The correct tiles were each diluted to 46 nM and stored in our repository.

### 4.3. Construction of Libraries: VersaTile Shuffling

Before performing the assembly reaction, all the OMPs, linkers, CBDs, and EADs per specific position were grouped, each in one tube per specific building block, by combining 1 µL of each tile (46 nM). The shuffling reaction comprised 1 μL of 25 nM pVTD2 (as destination vector), 1 μL of each tile mixture (on the required position), 10 U BsaI (as restriction enzyme), 3 U of T4 DNA ligase, and 2 μL of T4 DNA ligation buffer, with a total reaction volume of 20 μL. The VersaTile shuffling reaction mixture was incubated during 50 cycles of 2 min at 37 °C (restriction) and 3 min at 16 °C (ligation), followed by one cycle of 5 min at 50 °C (ligase inactivation) and one cycle of 5 min at 80 °C (BsaI inactivation). Afterwards, the entire reaction mixture was used for transformation of chemical competent BL21-CodonPlus(DE3)-RIL cells, followed by plating onto LB 1.5% agar plates, supplemented with 50 µg/mL kanamycin and 5% (*w*/*v*) sucrose.

### 4.4. High-Throughput Growth Inhibitory Assay

First, single colonies from the plated *E. coli* BL21-CodonPlus(DE3)-RIL cells from the VersaTile shuffling transformations were inoculated in a 96-deep well plate, filled with 500 μL LB supplemented with 50 µg/mL kanamycin in each well. The plate was incubated overnight (18 h) in a microtiter plate shaker (VWR) at 37 °C and 900 rpm, covered with tape allowing air exchange. The next day, the overnight culture was used to inoculate a new 96-deep well plate filled with 500 μL auto-induction medium and incubated for 5 h at 37 °C and 900 rpm in a microtiter plate shaker (VWR). Thereafter, the temperature was changed to 16 °C for 43 h. Following expression, the 96-deep well plate was centrifuged at 3200× *g* and 4 °C for 30 min, and the resulting supernatants are discarded. To release the recombinant lysin fusion proteins, cells were lysed by placing the deep-well plate over a chloroform-saturated filter, soaked in 80 mL chloroform, for 2 h, followed by 15 min evaporation of the residual chloroform. The obtained lysates in each well were suspended in 500 μL HEPES-NaOH, pH 7.4, and 1 U DNase I, followed by 1 h shaking at 700 rpm and room temperature to ensure complete resuspension. To confirm complete lysis, 5 μL of each well was spotted on an LB 1.5% agar plate and incubated overnight at 37 °C. The 96-deep well plate with lysates was centrifuged again to separate the soluble and insoluble fraction. The *Klebsiella* test strain was prepared in MH broth and incubated overnight at 37 °C. Subsequently, the culture was diluted in 2× MH to an OD_625nm_ of 0.08–0.1 (DeNovix^®^ DS-11 Spectrophotometer, Denovix Inc., Wilmington, NC, USA), followed by a 100-fold dilution. When mentioned, a final concentration of 1.0 mM EDTA-Na_2_ was added to the 100-fold diluted *Klebsiella* cell suspension. From this solution, 50 μL (5 × 10^6^ cells/mL) was added to each well of a microtiter plate together with 50 μL of the lysin variants, resulting in a 0.5 mM EDTA-Na_2_ concentration. The respective microtiter plates were incubated for 20 h at 37 °C. Finally, the growth inhibitory activity was determined using an endpoint measurement at 655 nm and the optical densities were converted to growth inhibition percentages. This parameter indicates the degree to which the bacterial growth is inhibited and is calculated with the following formula:

Growth inhibition (%) = ((1 − (OD_variant_ − OD_blank_))/(OD_negative control_ − OD_blank_)) × 100, (1)

with OD_variant_, OD_blank_ and OD_negative control_ corresponding to the optical density at 655 nm after 20 h for the variant, the medium, and the untreated cells respectively. Eventually, plasmids were extracted from top hits (>95%) of configuration 1 and 4, and the corresponding lysin sequences were analyzed using Sanger sequencing (LGC Genomics, Germany).

### 4.5. Statistics

For statistical analysis, we assumed a null hypothesis where, within every type of building block, every tile has an equal chance of occurring in an active design. This means that the probability of observing a tile is equal to one over the number of possible tiles for that position. To test whether a building block deviates from this uniform occurrence null hypothesis, we used a multinomial null distribution with uniform event probabilities to model the occurrence of tiles in an architecture. Our test statistic was the number of different tiles observed in the active design. We computed the *p*-value by taking 100,000 samples from the null distribution and counting the fraction that has fewer distinct tiles than in our observations. To test for significance of individual tiles within a position, we used a similar setup. The *p*-values were computed based on the implied binomial null distribution, i.e., the probability of seeing as many or more of the tiles in the active designs, given equal probabilities of occurrence. The corresponding *p*-values were compared to a significance level (α) of 0.1. Bonferroni correction was taken into consideration when multiple comparisons were performed (42 possible OMPs and 23 possible EADs at position 1 and 4 of configuration 1, and 6 possible linkers and 35 possible OMPs at position 3 and 4 of configuration 4, respectively). This was specifically applied in the case of the multiple dependent statistical test of the occurrences of the tiles per position [14].

To analyze if the occurrences of the flexible and rigid linkers after the screening differ significantly, a Chi-Square test was performed, using IBM^®^ SPSS^®^ Statistics software (IBM Corporation, New York, NY, USA, Version 26). The *p*-values are compared to a significance level (α) of 0.1 as well.

## Figures and Tables

**Table 1:** *p*-values calculated for the different distributions per position, both when EDTA is absent and present in the antibacterial assay. An asterisk indicates a significantly different distribution compared to a uniform distribution.

Configuration	Position	Type Building Block	*p*-Value (no EDTA)	*p*-Value (EDTA)
1	1	OMP	0.00035 *	0.0074 *
1	2	Linker	1	1
1	3	CBD	1	1
1	4	EAD	0.021 *	0.047 *
4	1	CBD	1	1
4	2	EAD	0.19677	0.39673
4	3	Linker	0.68365	0.04107 *
4	4	OMP	0.01894 *	0.00001 *

**Table 2:** Statistical analysis of overrepresented tiles. All significantly overrepresented tile types (Bonferroni correction, *p* < 0.1/42 (=0.0024) and *p* < 0.1/35 (=0.0029) for OMPs of configuration 1 and 4, respectively; *p* < 0.1/23 (=0.0043) for EADs of configuration 1; and *p* < 0.1/6 (=0.017) for the linker of configuration 4, with α =0.1) for a single or both conditions (without/with EDTA), are shown along with their number of occurrences and *p*-values. N° is the number of occurrences in the sequenced hits. The total numbers of sequenced hits per subgroup are as follows: N° (configuration 1, without EDTA) = max. 33, N° (configuration 1, with EDTA) = max. 36, N° (configuration 4, without EDTA) = max. 12, and N° (configuration 4, with EDTA) = max. 15. NS = not significant.

Configuration	Type Building Block	Tile	Without EDTA		With EDTA	
			N°	*p*-Value	N°	*p*-Value
1	OMP	Protegrin (OMP3)	7	8.4 × 10^−7^	10	4.8 × 10^−10^
1	OMP	Chrysophsin-1 (OMP20)	4	0.0010	3	NS (>0.0024)
1	EAD	201ϕ2-1gp229-EAD (EAD12)	5	0.0027	4	NS (>0.0043)
1	EAD	KZgp181 (EAD19)	3	NS (>0.0043)	7	0.00013
4	OMP	Cathelicidin-BF antimicrobial peptide (OMP35)	5	2.2 × 10^−7^	5	4.3 × 10^−6^
4	Linker	Rigid long helix (Linker 4)	4	NS (>0.017)	5	0.0080

## Data Availability

The data presented in this study are available within the article and the supplemental material.

## Supplementary Materials

The following are available online at https://www.mdpi.com/2079-6382/10/3/293/s1, Figure S1: Growth inhibition percentages of selected hits of configuration 1, Figure S2: Growth inhibition percentages of selected hits of configuration 4. Table S1: Primers designed for the construction of new linker tiles, Table S2: The total number of tiles per type of building block and per possible position, Table S3: Detailed description of each tile, Table S4: A list of the sequenced active constructs, supplemented with the sequences and numbers of their separate tiles and the growth inhibition percentages in absence and presence of EDTA. Table S5: A list of the sequenced active constructs of configuration 4, supplemented with the sequences and numbers of their separate tiles and the growth inhibition percentages in absence and presence of EDTA.
