# Supplementary material for: Rapid and High-Throughput Evaluation of Diverse Configurations of Engineered Lysins Using the VersaTile Technique
Source: Antibiotics (Basel). 2021 Mar 11;10(3):293. doi: 10.3390/antibiotics10030293 (PMC7998686; doi:10.3390/antibiotics10030293)
Supplement: Supplementary file 1 [file antibiotics-10-00293-s001.zip › Figure S1.pdf]

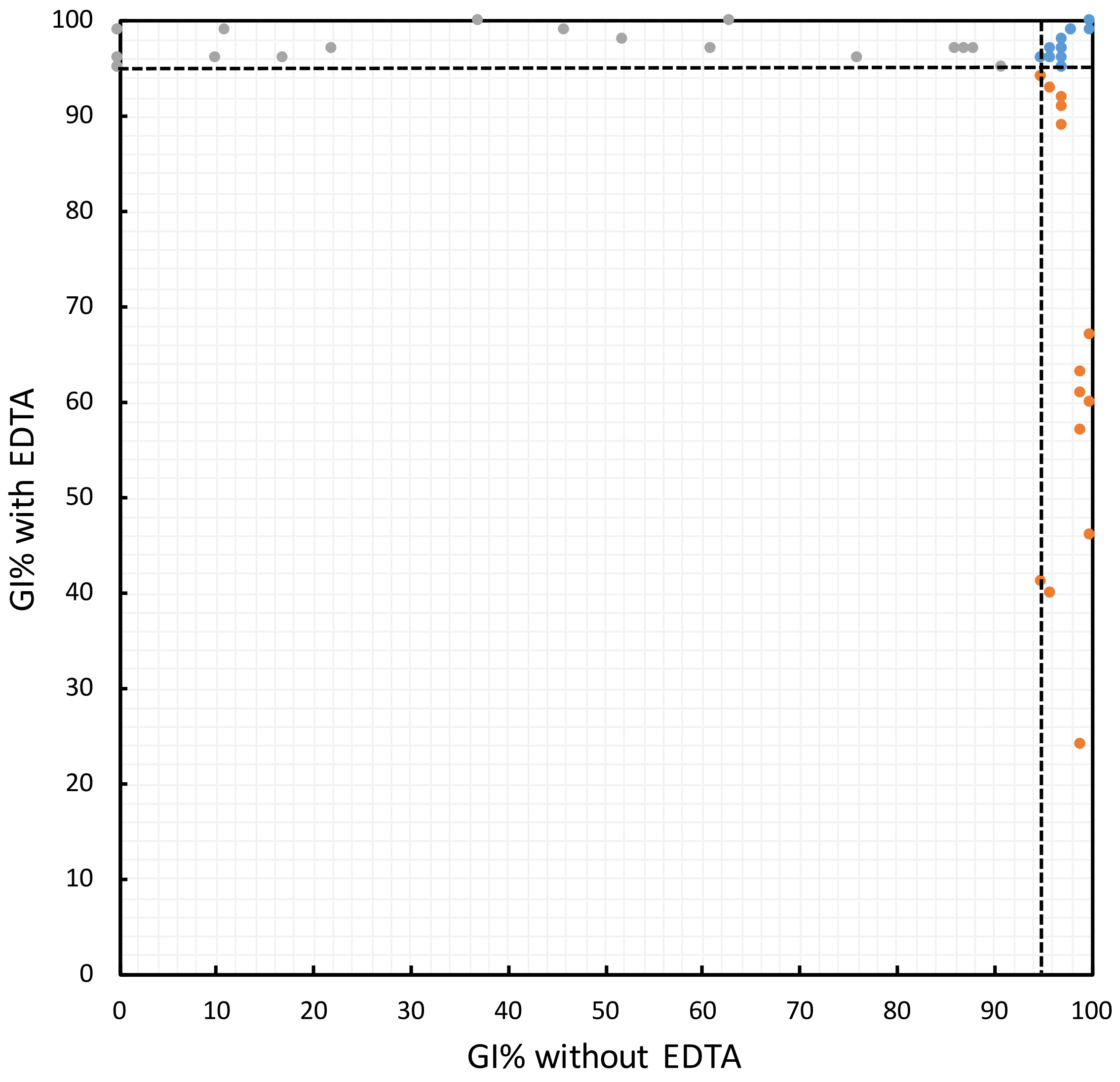

Figure S1. Growth inhibition percentages of selected hits of configuration 1. All selected hits from configuration 1 (n = 53) have at least 95% growth inhibition (GI%) either only in the absence of EDTA (orange; n = 17), the presence of EDTA (grey; n = 20) or in both the absence and presence of EDTA (blue; n = 16)
