# Supplementary material for: Rapid and High-Throughput Evaluation of Diverse Configurations of Engineered Lysins Using the VersaTile Technique
Source: Antibiotics (Basel). 2021 Mar 11;10(3):293. doi: 10.3390/antibiotics10030293 (PMC7998686; doi:10.3390/antibiotics10030293)
Supplement: Supplementary file 1 [file antibiotics-10-00293-s001.zip › Table S1.pdf]

Table S1. Primers designed for the construction of new linker tiles. The primers partially overlap and through hybridization they serve as template for the following step.

Linker	Primer sequence	
	**Forward**	**Reverse**
3	5′-CTGTCTCGTTTCTTCCACGCGGAACTG-3′	5′-CAGTTCCGCGTGGAAGAAACGAGACAG-3′
4	5′-GTTTTCAACCAGCGTAAAGAACACAAAGGTTACATGCTGGCG-3′	5′-CGCCAGCATGTAACCTTTGTGTTCTTTACGCTGGTTGAAAAC-3′
5	5′-ATCCCGCAGGGTCGTTCTCACCCGGTTCAGCCGTACCCGGGTGCGTTC-3′	5′-GAACGCACCCGGGTACGGCTGAACCGGGTGAGAACGACCCTGCGGGAT-3′
6	5′-CCGGCGGTTCCGCCGCCG-3′	5′-CGGCGGCGGAACCGCCGG-3′
7	5′-GAAGCGGCGGCGAAAGAAGCGGCGAAAGAAGCGGCGAAA-3′	5′-TTTCGCCGCTTCTTTCGCCGCTTCTTTCGCCGCCGCTTC-3′
