# Supplementary material for: Rapid and High-Throughput Evaluation of Diverse Configurations of Engineered Lysins Using the VersaTile Technique
Source: Antibiotics (Basel). 2021 Mar 11;10(3):293. doi: 10.3390/antibiotics10030293 (PMC7998686; doi:10.3390/antibiotics10030293)
Supplement: Supplementary file 1 [file antibiotics-10-00293-s001.zip › Table S2.pdf]

Table S2. The total number of tiles per type of building block and per possible position. The numbers correspond to the numbers in Table S3, which provides a more detailed description of each tile.

OMPs				Linkers		CBDs			EADs		
**Pos 1**	**Pos 2**	**Pos 3**	**Pos 4**	**Pos 2**	**Pos 3**	**Pos 1**	**Pos 2**	**Pos 3**	**Pos 2**	**Pos 3**	**Pos 4**
1	1		1	1	1	1	1	1	1	1	1
2	2		2	2			2	2		2	2
3	3	3	3	3	3	3	3	3	3	3	3
4	4	4	4	4	4		4	4		4	4
5	5		5	5	5	5	5	5		5	5
6	6	6	6	6	6	6	6	6	6	6	6
7	7	7	7	7	7				7	7	7
8	8	8	8							8	8
9		9	9						9	9	9
10	10	10	10						10	10	10
11	11	11	11							11	11
12	12	12	12						12	12	12
13	13	13	13						13	13	13
14	14	14	14						14	14	14
15			15						15	15	15
16	16	16	16						16	16	16
17			17						17	17	17
18	18	18	18							18	18
19			19							19	19
20										20	20
21	21	21	21							21	21
22	22	22	22							22	22
23	23	23	23							40	40
24		24									
25	25	25	25								
26	26	26	26								
27		27	27								
28	28	28	28								
29	29		29								
30	30	30									
31											
32	32		32								
33	33	33	33								
34											
35			35								
36			36								
37		37	37								
38											
39											
40		40	40								
41	41	41	41								
42	42	42	42								
