# Supplementary material for: Rapid and High-Throughput Evaluation of Diverse Configurations of Engineered Lysins Using the VersaTile Technique
Source: Antibiotics (Basel). 2021 Mar 11;10(3):293. doi: 10.3390/antibiotics10030293 (PMC7998686; doi:10.3390/antibiotics10030293)
Supplement: Supplementary file 1 [file antibiotics-10-00293-s001.zip › Table S3.pdf]

Table S3. Detailed description of each tile. The description and origin of the tile and the accession number and reference in literature are given, as well as the positions in the tile repository.

Code	Description	Origin	Accession number	Reference	Positions
OMP1	SMAP-29, Sheep myeloid antimicrobial peptide	Leukocytes of *Ovis aries*	AAA85470	https://doi.org/10.1016/S0014-5793(99)01600-2	1,2,4
OMP2	LL-37, Cathelicidin antimicrobial peptide preproprotein	Neutrophil specific granules of *Homo sapiens*	NP_004336	https://doi.org/10.1016/j.bbamem.2015.11.003	1,2,4
OMP3	Protegrin-1, Chain A	Leukocytes of *Sus scrofa*	AAB27599	https://doi.org/10.1016/S1074-5521(96)90145-3	1,2,3,4
OMP4	Indolicidin, Cathelicidin-4 precursor	Neutrophils of *Bos taurus*	NP_777252	https://doi.org/10.1016/j.bbrc.2003.08.095	1,2,3,4
OMP5	Magainin-2, Chain A	Skin of *Xenopus laevis*	2LSA_A	https://doi.org/10.3390/ijms19103041	1,2,4
OMP6	Cecropin P1	*Ascaris suum*	BAD89085	http://doi.org/10.1042/BJ20050218	1,2,3,4
OMP7	Cecropin A	*Aedes aegypti*	AAF59831	http://doi.org/10.1128/AAC.44.3.602-607.2000	1,2,3,4
OMP8	Pleurocidin precursor	Skin of *Pseudopleuronectes americanus*	P81941	https://doi.org/10.1038/mi.2013.37	1,2,3,4
OMP9	Buforin II	Stomach of *Bufo bufo*	AAB36002	https://doi.org/10.1006/bbrc.1998.8159	1,3,4
OMP10	Cecropin A2	*Drosophila melanogaster*	BAA28742	http://doi.org/10.1128/AAC.00686-17	1,2,3,4
OMP11	Sarcotoxin IA	Larva of *Sarcophaga peregrina*	AAA29988	https://doi.org/10.1016/S1046-5928(02)00697-6	1,2,3,4
OMP12	Nigrocin-2	Skin of *Pelophylax nigromaculatus*	P0C009	https://doi.org/10.1016/S0014-5793(01)02956-8	1,2,3,4
OMP13	Ascaphin-5	Skin of *Ascaphus truei*	P0CJ29	https://doi.org/10.1016/j.bbrc.2004.05.141	1,2,3,4
OMP14	Ranalexin	Skin of *Rana catesbeiana*	P39084	http://www.jbc.org/content/269/14/10849.long	1,2,3,4
OMP15	Pseudin-1	Skin of *Pseudis paradoxa*	P83188	https://doi.org/10.1006/bbrc.2001.5884	1,4
OMP16	Sushi 1, Derived from factor C	*Carcinoscorpius rotundicauda*	AAB34362	https://doi.org/10.1007/s00018-008-7456-0	1,2,3,4
OMP17	Melittin	Venom of *Apis mellifera*	670043A	https://doi.org/10.3892/mmr.2015.4275	1,4

OMP18	TP4, Tilapia piscidin 4	Gills of *Oreochromis niloticus*	5H2S_A	https://doi.org/10.1016/j.cbpb.2008.12.018	1,2,3,4
OMP19	Ci-MAM-A24, Molecule against microbes A	Haemocytes of *Ciona intestinalis*	ACA97856	https://doi.org/10.1042/BJ20080398	1,4
OMP20	Chrysophsin-1	Gills of *Pagrus major*	P83545	https://doi.org/10.1046/j.1432-1033.2003.03419.x	1
OMP21	Piscidin-like peptide	Spleen of *Larimichthys crocea*	ACE78289	https://www.ncbi.nlm.nih.gov/pmc/articles/PMC5359322/	1,2,3,4
OMP22	Myxinidin	Derived from epidermal mucus of *Myxine glutinosa* L.	Protein sequence: GIHDILKYGKPS	https://doi.org/10.1007/s10126-009-9189-y	1,2,3,4
OMP23	PAM2, *Platypus* cathelicidin peptide	Identified in *Platypus* genome using bioinformatics	Protein sequence: RPWAGNGSVHRYTVLSPRLKTQ	https://doi.org/10.1371/journal.pone.0024030	1,2,3,4
OMP24	NRC-13, Pleurocidin-like peptide AP3	*Hippoglossoides platessoides*	AAP55795	https://doi.org/10.1128/AAC.47.8.2464-2470.2003	1,3
OMP25	3IQ2, Synthetic construct	Human cytoskeletal scaffolding proteins IQGAP3	Protein sequence: TWLPAVIKIQAHWRGYRQRKIYL	https://doi.org/10.1016/j.biochi.2012.12.004	1,2,3,4
OMP26	IR2, Synthetic construct	Protegrin-1	Protein sequence: IRIRCRRRFCRIRI	https://doi.org/10.1016/j.biomaterials.2014.06.005	1,2,3,4
OMP27	FR2, Synthetic construct	Protegrin-1	Protein sequence: FRFRCRRRFCRFRF	https://doi.org/10.1016/j.biomaterials.2014.06.005	1,3,4
OMP28	CEM1, Synthetic construct	MSI-78 (AWS34388)	Protein sequence: GIGKFLKKAKKF	https://doi.org/10.1016/j.bbamem.2015.02.001	1,2,3,4
OMP29	LfcinB, Chain A, Lactoferricin	*Bos taurus*	1LFC_A	https://doi.org/10.1021/bi972323m	1,2,4
OMP30	NRC-16, Pleurocidin-like peptide GC3.8	*Glyptocephalus cynoglossus*	AAP55799	https://doi.org/10.1128/AAC.47.8.2464-2470.2003	1,2,3
OMP31	TB_KKG6A, Synthetic construct	Chain A, temporinB_KKG6A (6GIJ_A)	Protein sequence: KKLLPIVANLLKSLL	https://doi.org/10.1038/s41598-018-37630-3	1
OMP32	Dermaseptin S4L7KA14K, Dermaseptin-4	Skin of *Phyllomedusa sauvagii*	P80280	https://doi.org/10.1128/AAC.46.3.689-694.2002	1,2,4
OMP33	Piscidin 119K, Chain A, Moronecidin	Mast cells of	2JOS_A	https://doi.org/10.1021/bi0620297	1,2,3,4

		Morone saxatilis			
OMP34	Fowlicidin-2	*Gallus gallus*	ADZ99029	https://doi.org/10.1002/btpr.2041	1
OMP35	Cathelicidin-BF antimicrobial peptide	Snake venom of *Bungarus fasciatus*	B6D434	https://doi.org/10.1371/journal.pone.0003217	1,4
OMP36	E7K_I9K_IsCT, Chain A, Cytotoxic linear peptide IsCT	Scorpion venom of *Opisthacanthus madagascariensis*	1T52_A	https://doi.org/10.1016/j.bbrc.2004.08.144	1,4
OMP37	PCNP, Polycationic nonapeptide, Synthetic construct	*In silico* design	Protein sequence:<br><br>KRKKRKKRK	https://doi.org/10.1128/mBio.01379-14	1,3,4
OMP38	E7K_ISCT, Chain A, Cytotoxic linear peptide IsCT	Scorpion venom of *Opisthacanthus madagascariensis*	1T52_A	https://doi.org/10.1016/j.bbrc.2004.08.144	1
OMP39	Dermcidin	Sweat glands of humans	ABQ53649.1	https://doi.org/10.1038/ni732	1
OMP40	Bac7	Bovine neutrophils	Protein sequence:<br><br>RRIRPRPPRLPRPRPRPLPFPRPG PRPIPRPLPFP	https://doi.org/10.1021/jm501367p	1,3,4
OMP41	Epinecidin	*Epinephelus coioides*	APM86638.1	https://dx.doi.org/10.3389%2Ffmicb.2019.02631	1,2,3,4
OMP42	SSL-25, dermcidin-derived peptide	Human sweat	Protein sequence:<br><br>SSLLEKGLDGAKKAVGGLG KLGKDA	https://doi.org/10.1016/j.bbamem.2017.09.004	1,2,3,4
Link1	Flexible short linker	*In silico* design	Protein sequence: AGAGAG	/	1,2
Link2	Flexible median linker	*In silico* design	Protein sequence: GAGAGAGAGAGAGA	/	1
Link3	Rigid short helix	*In silico* design	Protein sequence:<br><br>LSRFFHAEL	/	1,2
Link4	Rigid long helix	*In silico* design	Protein sequence:<br><br>VFNQRKEHKGYMLA	/	1,2
Link5	Rigid long coil	*In silico* design	Protein sequence:<br><br>IPQGRSHPVQPYPGAF	/	1,2

Link6	Rigid short coil	*In silico* design	Protein sequence: PAVPPP	/	1,2
Link7	Rigid, optimal helix	*In silico* design	Protein sequence: EAAAKEAAKEAAK	/	1,2
CBD1	KZ144-CBD, CBD of endolysin KZ144	*Pseudomonas* phage phiKZ	NP_803710	https://doi.org/10.1006/jmbi.2001.5396	1,2,3
CBD2	EL188-CBD, CBD of endolysin EL188	*Pseudomonas* phage EL	YP_418221	https://doi.org/10.1016/j.jmb.2005.08.075	2,3
CBD3	PVP-SE1gp146-CBD, CBD of endolysin PVP-SE1	*Salmonella phage* PVP-SE1	YP_004893952	https://doi.org/10.1128/JVI.01769-10	1,2,3
CBD4	OBPgp279-CBD, CBD of endolysin OBP	*Pseudomonas* phage OBP	YP_004958186	https://doi.org/10.1128/JVI.06330-11	2,3
CBD5	201phi2-1gp229-CBD, CBD of endolysin 201phi2-1	*Pseudomonas* phage 201phi2-1	YP_001956952	https://doi.org/10.1016/j.virol.2008.04.004	1,2,3
CBD6	KZ144-CBD w/ser	*Pseudomonas* phage phiKZ	NP_803710	https://doi.org/10.1006/jmbi.2001.5396	1,2,3
EAD1	PsP3gp10, EAD of endolysin PsP3	*Salmonella* phage PsP3	NP_958065	https://doi.org/10.1016/0042-6822(91)90573-T	1,2,3
EAD2	P2gp09, EAD of endolysin P2gp09	*Escherichia* phage P2	NP_046765	https://doi.org/10.1080/21597081.2016.1145782	2,3
EAD3	K11gp3.5, EAD of endolysin K11	*Enterobacteria* phage K11	AAX62800	https://doi.org/10.1016/j.pep.2005.03.026	1,2,3
EAD4	CR8gp3.5, EAD of endolysin CR8	*Citrobacter* phage CR8	YP_009004176	https://doi.org/10.1128/genomeA.00146-14	2,3
EAD5	LUZ24gp67, EAD of endolysin LUZ24	*Pseudomonas* phage LUZ24	YP_001671940	https://doi.org/10.1016/j.virol.2008.04.038	2,3
EAD6	BcepC6Bgp22, EAD of endolysin BcepC6B	*Burkholderia* phage BcepC6B	YP_024942	Uniprot: AY605181	1,2,3
EAD7	LysEC8, EAD of endolysin phAPEC8	*Escherichia* phage phAPEC8	YP_007348465	https://doi.org/10.1128/JVI.02374-12	1,2,3
EAD8	KZ144-EAD, EAD of endolysin KZ144	*Pseudomonas* phage phiKZ	NP_803710	https://doi.org/10.1006/jmbi.2001.5396	2,3
EAD9	EL188-EAD, EAD of endolysin EL	*Pseudomonas* phage EL	YP_418221	https://doi.org/10.1016/j.jmb.2005.08.075	1,2,3
EAD10	PVP-SE1gp146-EAD, EAD of endolysin PVP-SE1	*Salmonella* phage PVP-SE1	YP_004893952	https://doi.org/10.1128/JVI.01769-10	1,2,3

EAD11	OBPgp279-EAD, EAD of endolysin OBP	*Pseudomonas* phage OBP	YP_004958186	https://doi.org/10.1128/JVI.06330-11	2,3
EAD12	201Phi2-1gp229-EAD, EAD of endolysin *201phi2-1*	*Pseudomonas* phage 201phi2-1	YP_001956952	https://doi.org/10.1016/j.virol.2008.04.004	1,2,3
EAD13	Acibel007, EAD of endolysin *vB_AbaP_Acibel007*	*Acinetobacter* phage vB_AbaP_Acibel007	YP_009103259	https://doi.org/10.1371/journal.pone.0104853	1,2,3
EAD14	vB_PsyM_KIL1gp019, EAD of endolysin *vB_PsyM_KIL1*	*Pseudomonas* phage vB_PsyM_KIL1	YP_009276009	https://doi.org/10.3389/fmicb.2016.00279	1,2,3
EAD15	XccLys, EAD of endolysin Xcc	*Xanthomonas campestris* pv. *campestris* phage	Unpublished sequence	-	1,2,3
EAD16	Shivanigp41, EAD of endolysin *Shivani*	*Salmonella* phage Shivani	YP_009194685.1	https://doi.org/10.1128/genomeA.01443-14	1,2,3
EAD17	Vpept, EAD of endolysin *VvAW1*	*Vibrio* phage VvAW1	YP_007518361	https://doi.org/10.4056/sigs.2846206	1,2,3
EAD18	KMVgp36C, VAPGH of phiKMV	*Pseudomonas* phage phiKMV	NP_877475	https://doi.org/10.1099/mic.0.28431-0	2,3
EAD19	KZgp181, VAPGH of phiKZ	*Pseudomonas* phage phiKZ	AAL83082	https://doi.org/10.1006/jmbi.2001.5396	2,3
EAD20	BcepC6Bgp16, VAPGH of BcepC6B	*Burkholderia* phage BcepC6B	YP_024936	Uniprot: AY605181	2,3
EAD21	OBPgp276, VAPGH of OBP	*Pseudomonas* phage OBP	YP_004958183	https://doi.org/10.1128/JVI.06330-11	2,3
EAD22	KP32gp15	*Klebsiella* phage KP32	YP_003347533.1	https://doi.org/10.1007/s00253-012-4294-7	2,3
EAD40	OBPgp149	*Pseudomonas fluorescens* phage	YP_004958056.1	https://doi.org/10.1128/jvi.06330-11	2,3
