# Supplementary material for: Rapid and High-Throughput Evaluation of Diverse Configurations of Engineered Lysins Using the VersaTile Technique
Source: Antibiotics (Basel). 2021 Mar 11;10(3):293. doi: 10.3390/antibiotics10030293 (PMC7998686; doi:10.3390/antibiotics10030293)
Supplement: Supplementary file 1 [file antibiotics-10-00293-s001.zip › Table S4.pdf]

Table S4. A list of the sequenced active constructs of configuration 1, supplemented with the sequences and numbers of their separate tiles and the growth inhibition percentages in absence and presence of EDTA.

Nr	OMP	Linker	CBD	EAD	OMP	Linker	CBD	EAD	GI% without EDTA	GI% with EDTA
1	Cathelicidin-BF	Rigid, optimal helix	KZ144-CBD	EL188-EAD	OMP35	link7	CBD1	EAD9	97	98
2	Cathelicidin-BF	Rigid short coil	EL188-CBD	KZgp181	OMP35	link6	CBD2	EAD19	0	99
3	Cathelicidin-BF	Rigid, optimal helix	KZ144-CBD	KZgp181	OMP35	link7	CBD1	EAD19	100	99
4	Cecropin A (D. melanogaster)	Flexible median linker	PVP-SE1gp146-CBD	EL188-EAD	OMP10	link2	CBD3	EAD9	95	41
5	Cecropin P1	Rigid short coil	PVP-SE1gp146-CBD	201j2-1gp229-EAD	OMP6	link6	CBD3	EAD12	97	96
6	CecropinA(A.aegypti)A	Rigid long helix	201phi2-1gp229-CBD	XccBgp35	OMP7	link4	CBD5	EAD15	97	91
7	Chrysophsin-1	Rigid short coil	OBPgp279-CBD	Phage2b.1bgp019	OMP20	link6	CBD4	EAD14	96	96
8	Chrysophsin-1	Rigid short coil	OBPgp279-CBD	Phage2b.1bgp019	OMP20	link6	CBD4	EAD14	96	96
9	Chrysophsin-1	Rigid short coil	EL188-CBD	PsP3gp10	OMP20	link6	CBD2	EAD1	96	40
10	Chrysophsin-1	Rigid short helix	KZ144-CBD	Phage2b.1bgp019	OMP20	link3	CBD1	EAD13	98	99
11	E7K_I9K_IsCT	Flexible median linker	KZ144-CBD	201j2-1gp229-EAD	OMP36	link2	CBD1	EAD12	97	95
12	Epinecidin	Rigid short helix	EL188-CBD	KZ144-EAD	OMP41	link3	CBD2	EAD8	61	97
13	Fowlicidin-2	Rigid short coil	PVP-SE1gp146-CBD	EL188-EAD	OMP34	link6	CBD3	EAD9	37	100
14	Fowlicidin-2	Rigid short coil	201phi2-1gp229-CBD	BcepC6Bgp16	OMP34	link6	CBD5	EAD20	99	57
15	IR2	Rigid short coil	KZ144-CBD	BcepC6Bgp16	OMP26	link4	CBD1	EAD20	95	94
16	IR2	Rigid short coil	KZ144-CBD	KZgp181	OMP26	link6	CBD1	EAD19	63	100
17	LfcinB	Rigid short coil	PVP-SE1gp146-CBD	LUZ24gp67	OMP29	link6	CBD3	EAD5	52	98
18	LfcinB	Rigid short coil	EL188-CBD	LUZ24gp67	OMP29	link6	CBD2	EAD5	76	96
19	LL-37	Flexible median linker	EL188-CBD	KMVgp36C	OMP2	link2	CBD2	EAD18	17	96
20	Magainin	Rigid short coil	OBPgp279-CBD	CR8gp3.5	OMP5	link6	CBD4	EAD4	22	97
21	Magainin	Rigid long helix	OBPgp279-CBD	201j2-1gp229-EAD	OMP5	link4	CBD4	EAD12	97	97
22	Magainin	Rigid long helix	201phi2-1gp229-CBD	KMVgp36C	OMP5	link4	CBD5	EAD18	97	97
23	Melittin	Rigid long coil	KZ144-CBD w/Ser	KZgp181	OMP17	link5	CBD6	EAD19	100	100
24	Melittin	Rigid short helix	KZ144-CBD w/Ser	EL188-EAD	OMP17	link3	CBD6	EAD9	99	24
25	Nigrocine 2	Rigid short coil	201phi2-1gp229-CBD	Phage2b.1bgp019	OMP12	link6	CBD5	EAD14	97	97
26	NRC-13	Rigid, optimal helix	EL188-CBD	KZ144-EAD	OMP24	link7	CBD2	EAD8	95	94

27	NRC-13	Flexible short linker	EL188-CBD	CR8gp3.5	OMP24	link1	CBD2	EAD4	96	93
28	PAM2	Rigid long coil	PVP-SE1gp146-CBD	KZgp181	OMP23	link5	CBD3	EAD19	46	99
29	Protegrin	Rigid long helix	OBPgp279-CBD	PVP-SE1gp146-EAD	OMP3	link4	CBD4	EAD10	0	96
30	Protegrin	Flexible short linker	KZ144-CBD	KZgp181	OMP3	link1	CBD1	EAD19	0	95
31	Protegrin	Rigid short coil	EL188-CBD	OBPgp279-EAD	OMP3	link6	CBD2	EAD11	0	95
32	Protegrin	Rigid long coil	KZ144-CBD	PVP-SE1gp146-EAD	OMP3	link5	CBD1	EAD10	10	96
33	Protegrin	Rigid short coil	PVP-SE1gp146-CBD	LUZ24gp67	OMP3	link6	CBD3	EAD5	100	46
34	Protegrin	Rigid long helix	PVP-SE1gp146-CBD	Phage003/011gp040	OMP3	link4	CBD3	EAD16	11	99
35	Protegrin	Rigid short coil	PVP-SE1gp146-CBD	EL188-EAD	OMP3	link6	CBD3	EAD9	87	97
36	Protegrin	Flexible median linker	OBPgp279-CBD	PVP-SE1gp146-EAD	OMP3	link2	CBD4	EAD10	88	97
37	Protegrin	Flexible median linker	KZ144-CBD	PVP-SE1gp146-EAD	OMP3	link2	CBD1	EAD10	91	95
38	Protegrin	Rigid, optimal helix	KZ144-CBD	Phage2b.1bgp019	OMP3	link7	CBD1	EAD14	95	96
39	Protegrin	Flexible median linker	PVP-SE1gp146-CBD	KZgp181	OMP3	link2	CBD3	EAD19	95	94
40	Protegrin	Rigid, optimal helix	201phi2-1gp229-CBD	Phage2b.1bgp019	OMP3	link7	CBD5	EAD15	96	40
41	Protegrin	Flexible median linker	PVP-SE1gp146-CBD	BcepC6Bgp16	OMP3	link2	CBD3	EAD20	97	95
42	Protegrin	Rigid long coil	PVP-SE1gp146-CBD	EL188-EAD	OMP3	link5	CBD3	EAD9	97	92
43	Protegrin	Rigid long coil	201phi2-1gp229-CBD	LUZ24gp67	OMP3	link5	CBD5	EAD5	99	63
44	Pseudin 1	Rigid long helix	201phi2-1gp229-CBD	KZ144-EAD	OMP15	link4	CBD5	EAD8	100	60
45	Ranalexin	Rigid long helix	EL188-CBD	KMVgp36C	OMP14	link4	CBD2	EAD18	98	99
46	Ranalexin	Rigid long helix	201phi2-1gp229-CBD	201j2-1gp229-EAD	OMP14	link4	CBD5	EAD12	99	61
47	SMAP-29	Rigid long helix	201phi2-1gp229-CBD	OBPgp149	OMP1	link4	CBD5	EAD40	100	67
48	SSL-25	Rigid, optimal helix	OBPgp279-CBD	LUZ24gp67	OMP42	link7	CBD4	EAD5	97	89
49	Sushi 1	Rigid, optimal helix	201phi2-1gp229-CBD	LUZ24gp67	OMP16	link7	CBD5	EAD5	0	99
50	Sushi 1	Rigid short coil	KZ144-CBD w/Ser	KZgp181	OMP16	link6	CBD6	EAD19	0	96
51	TB_KKG6A	Rigid short coil	201phi2-1gp229-CBD	LUZ24gp67	OMP31	link6	CBD5	EAD5	86	97
52	TB_KKG6A	Flexible median linker	PVP-SE1gp146-CBD	OBPgp149	OMP31	link2	CBD3	EAD40	95	96
53	TP4	Rigid long helix	KZ144-CBD	201j2-1gp229-EAD	OMP18	link4	CBD1	EAD12	96	97
