# Supplementary material for: Rapid and High-Throughput Evaluation of Diverse Configurations of Engineered Lysins Using the VersaTile Technique
Source: Antibiotics (Basel). 2021 Mar 11;10(3):293. doi: 10.3390/antibiotics10030293 (PMC7998686; doi:10.3390/antibiotics10030293)
Supplement: Supplementary file 1 [file antibiotics-10-00293-s001.zip › Table S5.pdf]

Table S5. A list of the sequenced active constructs of configuration 4, supplemented with the sequences and numbers of their separate tiles and the growth inhibition percentages in absence and presence of EDTA.

Nr	CBD	EAD	Linker	OMP	CBD	EAD	Linker	OMP	GI% without EDTA	GI% with EDTA
1	PVP-SE1gp146-CBD	EL188-EAD	Flexible short linker	Sarcotoxin IA	CBD3	EAD9	link1	OMP11	98	95
2	201phi2-1gp229-CBD	XccLys	Rigid long helix	Sarcotoxin IA	CBD5	EAD15	link4	OMP11	96	67
3	PVP-SE1gp146-CBD	vB_PsyM_KIL1gp019	Flexible short linker	Sushi 1	CBD3	EAD14	link1	OMP16	96	56
4	KZ144-CBD	XccLys	Flexible short linker	TP4	CBD1	EAD15	link1	OMP18	95	87
5	KZ144-CBD	XccLys	Flexible short linker	TP4	CBD1	EAD15	link1	OMP18	92	95
6	201phi2-1gp229-CBD	K11gp3.6	Rigid short coil	Piscidin-like peptide	CBD5	EAD3	link6	OMP21	38	98
7	KZ144-CBD	Acibel007	Rigid long coil	3IQ2	CBD1	EAD13	link5	OMP25	95	97
8	KZ144-CBD w/ser	PVP-SE1gp146-EAD	Rigid long coil	PAM2	CBD6	EAD10	link5	OMP23	65	97
9	PVP-SE1gp146-CBD	K11gp3.5	Rigid long helix	SSL-25	CBD3	EAD3	link4	OMP42	97	98
10	KZ144-CBD	EL188-EAD	Flexible short linker	FR2	CBD1	EAD9	link1	OMP27	15	96
11	201phi2-1gp229-CBD	vB_PsyM_KIL1gp019	Rigid short helix	LfcinB	CBD5	EAD14	link3	OMP29	59	97
12	KZ144-CBD	XccLys	Rigid long helix	Indolicidin	CBD1	EAD15	link4	OMP4	95	97
13	PVP-SE1gp146-CBD	Acibel007	Rigid long helix	Dermaseptin S4L7KA14K	CBD3	EAD13	link4	OMP32	93	98
14	KZ144-CBD	PsP3gp10	Rigid long coil	Cathelicidin-BF antimicrobial peptide	CBD1	EAD1	link5	OMP35	90	96
15	KZ144-CBD	PVP-SE1gp146-EAD	Flexible short linker	Cathelicidin-BF antimicrobial peptide	CBD1	EAD10	link1	OMP35	95	98
16	201phi2-1gp229-CBD	EL188-EAD	Rigid long helix	Cathelicidin-BF antimicrobial peptide	CBD5	EAD9	link4	OMP35	96	94
17	PVP-SE1gp146-CBD	EL188-EAD	Rigid long coil	Cathelicidin-BF antimicrobial peptide	CBD3	EAD9	link5	OMP35	36	97
18	KZ144-CBD w/ser	Acibel007	Rigid long coil	Cathelicidin-BF antimicrobial peptide	CBD6	EAD13	link5	OMP35	97	38
19	201phi2-1gp229-CBD	vB_PsyM_KIL1gp019	Rigid long coil	Cathelicidin-BF antimicrobial peptide	CBD5	EAD14	link5	OMP35	96	96
20	KZ144-CBD w/ser	Vpept	Rigid long helix	Cathelicidin-BF antimicrobial peptide	CBD6	EAD17	link4	OMP35	95	58

21	201phi2-1gp229-CBD	K11gp3.7	Rigid long helix	Cathelicidin-BF antimicrobial peptide	CBD5	EAD3	link4	OMP35	65	95
